# Transcriptomic Analysis of the Spleen of Different Chicken Breeds Revealed the Differential Resistance of *Salmonella* Typhimurium

**DOI:** 10.3390/genes13050811

**Published:** 2022-05-02

**Authors:** Mohamed Shafey Elsharkawy, Hailong Wang, Jiqiang Ding, Mahmoud Madkour, Qiao Wang, Qi Zhang, Na Zhang, Qinghe Li, Guiping Zhao, Jie Wen

**Affiliations:** 1 State Key Laboratory of Animal Nutrition, Institute of Animal Sciences, Chinese Academy of Agricultural Sciences, Beijing 100193, China; shafey.nrc@hotmail.com (M.S.E.); wanghailong_94@163.com (H.W.); dingjq2020@126.com (J.D.); wangqiao01@caas.cn (Q.W.); Zhangq0117@126.com (Q.Z.); Zhangna4520@163.com (N.Z.); liqinghe@caas.cn (Q.L.); zhaoguiping@caas.cn (G.Z.); 2 Animal Production Department, National Research Centre, Dokki, Cairo 12622, Egypt; mahmoud.madkour9@gmail.com

**Keywords:** spleen, transcriptome, *Salmonella* Typhimurium, immune-related genes and pathway, Beijing-You, cobb chicken

## Abstract

*Salmonella* Typhimurium (ST) is a foodborne pathogen that adversely affects the health of both animals and humans. Since poultry is a common source and carrier of the disease, controlling ST infection in chickens will have a protective impact on human health. In the current study, Beijing-You (BY) and Cobb chicks (5-day-old specific-pathogen-free) were orally challenged by 2.4 × 10^12^ CFU ST, spleen transcriptome was conducted 1 day post-infection (DPI) to identify gene markers and pathways related to the immune system. A total of 775 significant differentially expressed genes (DEGs) in comparisons between BY and Cobb were identified, including 498 upregulated and 277 downregulated genes (fold change ≥2.0, *p* < 0.05). Several immune response pathways against *Salmonella* were enriched, including natural killer-cell-mediated-cytotoxicity, cytokine–cytokine receptor interaction, antigen processing and presentation, phagosomes, and intestinal immune network for IgA production, for both BY and Cobb chickens. The BY chicks showed a robust response for clearance of bacterial load, immune response, and robust activation of phagosomes, resulting in ST resistance. These results confirmed that BY breed more resistance to ST challenge and will provide a better understanding of BY and Cobb chickens’ susceptibility and resistance to ST infection at the early stages of host immune response, which could expand the known intricacies of molecular mechanisms in chicken immunological responses against ST. Pathways induced by *Salmonella* infection may provide a novel approach to developing preventive and curative strategies for ST, and increase inherent resistance in animals through genetic selection.

## 1. Introduction

*Salmonella* Typhimurium (ST) is a gram-negative intestinal pathogen that doesn’t cause serious morbidity or death; however, chickens can carry this bacterium for several weeks without showing any symptoms, thus posing a serious public health risk [1,2,3]. Recently, ST became the most prevalent serotype in eggs and meat produced in China [4,5,6]. ST can be difficult to treat in humans and animals because it is often very resistant to several antibiotics, such as nalidixic acid, trimethoprim, and sulfamethoxazole [7,8,9]. *Salmonella* typically infects chickens through the fecal–oral route. The innate immune system in particular is important for *Salmonella* infections, as adaptive immunity is not yet developed in young chickens. [10]. Therefore, understanding host immune response mechanisms and resistance to ST infection is crucial for reducing economic losses in chicken productions and for protecting animal and human health [11,12]. Beijing-You (BY) is a local Chinese chicken breed that is more resistant to stress and disease than White Leghorn [13]. Candidate SNPs linked to immunological features were identified in BY chicken by a genome-wide association study (antibody responses against SRBC and AIV, serum IgY level, and heterophils and lymphocytes numbers and ratios) [14]. Resistance to *Salmonella* Pullorum is higher in BY chickens than in Rhode Island Red chickens [15]. Antibody titers raised for Newcastle disease and avian influenza vaccinations were higher in BY chickens than White Leghorn chickens [16]. During *Salmonella* infection, the spleen plays a vital role in revealing cellular damage and pathogenic mechanisms. In addition, mounting data shows that the spleen in avian species is more responsible for innate response shortly after disease detection by scavenging antigens from the blood than in mammalian species [17,18]. Little is known about the immune system genes and signaling pathways in BY and Cobb chickens during ST infection. This study identifies pathways and genes differentially expressed in BY compared to Cobb chickens post-ST challenging to elucidate effective host resistance mechanisms against ST.

## 2. Materials and Methods

### 2.1. Animals and Experimental Diets

The Animal Ethics Committee of the Institute of Animal Sciences, Chinese Academy of Agricultural Sciences (IAS-CAAS, Beijing, China) approved the experimental procedures (IASCAAS2021-31). A total of 100 one-day-old Cobb broiler chicks purchased from Poultry Breeding Co., Ltd. (Beijing, China) and 100 one-day-old BY chicks obtained from the Changping Experimental farm of Institute of Animal Sciences (Beijing, China) were maintained under strict hygienic conditions in two isolated control chamber rooms at the experimental center of China Agricultural University (Beijing, China).

The *Salmonella* Typhimurium strain was resuscitated overnight in Luria–Bertani (LB) broth (Amresco, Washington, DC, USA) at 37 °C in an orbital shaking incubator at 150 rpm. After recovery, ST was cultured for 12 h and concentrated in a centrifuge. The final number of colony-forming units (CFU) was determined by plating serial dilutions as described in previous studies [19]. *Salmonella* testing was performed on all the chicks [20], and the positive chicks were eliminated. At 3 d of age, all the birds were challenged orally with 1 mL PBS holding 2.4 × 10^12^ CFU ST. At 1 DPI, the spleens and livers were collected. The spleens were placed in liquid nitrogen for storage, and the livers were used to measure the bacterial count of ST by calculating the number of colony-forming units on the MacConkey agar. According to the liver bacterial count and symptoms, (i.e., drooping wings, dying, and diarrhea), the spleens of 20 chicks from each group were chosen for RNA sequencing.

### 2.2. RNA Extraction, cDNA Library Preparation, and RNA-seq

The spleen RNAs were extracted from the 40 chicks using a QIAGEN RNeasy Kit (Qiagen, Hilden, Germany). The quality and quantity of the total RNA were assessed by a 2100 Bioanalyzer and RNA 6000 Nano kit (Agilent, Santa Clara, CA, USA). For the mRNA library construction and deep sequencing, 3 μg total of RNA was prepared using the TruSeq RNA Sample Preparation Kit (Illumina, San Diego, CA, USA) to capture the coding transcriptome. After purification, the RNA was fragmented by using divalent cations at 95 °C. The cleaved RNA fragments were reversely transcribed into first-strand cDNA using TruSeq RNA Library Preparation Kit, followed by second-strand cDNA synthesis. After cDNA fragment purification and adaptor ligation, RNA sequencing was performed on the HiSeq X Ten platform (Illumina, San Diego, CA, USA). 

Sequence data can be accessed at the GSA of BIG Data Center, Beijing Institute of Genomics (BIG), Chinese Academy of Sciences and will be publicly accessible at https://ngdc.cncb.ac.cn/gsa/ (access number: CRA006334, accessed on 11 March 2024).

### 2.3. Differentially Expressed Genes and Function Enrichment Analysis

The quality control of reads were performed using FastQC (v0.11.5) [21]. Sequence adapters and low-quality reads were eliminated. Hisat2 was used mapping the sequencing data to the chicken reference genome (Gallus gallus 6.0) [22]. The expression quantities of the mapped transcripts were calculated using Htseq [23]. The DEGs analyses were conducted by DESeq2 [24]. Genes with fold change ≥2 and padj < 0.5 were considered DEGs. To assess the variation between samples, a cluster map of DEGs was conducted by pheatmap (https://CRAN.R-project.org/package=pheatmap (accessed on 15 April 2021)). To provide an overview of the DEGs, volcano plots were performed by ggplot2 [25]. The transcripts were converted to Entrez Gene IDs, and the gene enrichment analysis based on the DEGs was conducted by Gene Ontology (GO) functional enrichment analysis [26].

## 3. Results

### 3.1. Overview of the Bacterial Load between BY and Cobb Chicks

**Figure 1:**
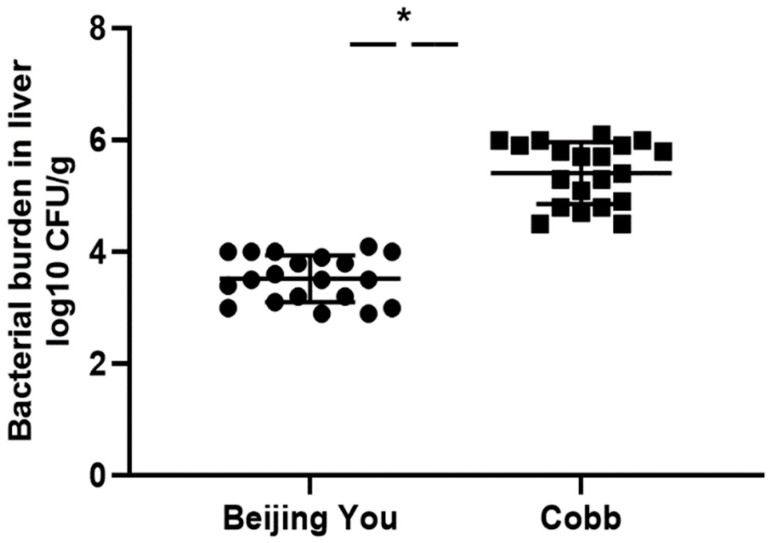
Bacterial burden in the liver 1 day after oral infection of chicks with 2.4 × 10^12^ CFU ST/chick. The results are presented as individual values, mean ± standard deviation, * *p* < 0.05.

The number of colonies on MacConkey agar was calculated to assess the bacterial burden in the liver. As seen in Figure 1, BY chickens showed significantly lower liver bacterial count than Cobb chickens, indicating that BY chicks were more effective in eliminating ST. These findings explain the severe symptoms that appeared in Cobb more than BY chicks, which might illustrate the higher resistance against *Salmonella* shown in BY chickens than in Cobb chickens.

### 3.2. Sequencing of Spleen Transcriptomes

RNA-Seq of the spleen produced >20 Mb clean reads of 40 samples. Approximately 93% of the clean reads had quality scores exceeding the Q30 value. The data proved the reliability of the RNA-seq and could be used for data analysis. After removing the interference reads, the clean reads represented >93%, as shown in Supplementary Table S1.

### 3.3. DEGs in Response to ST Infection

**Figure 2:**
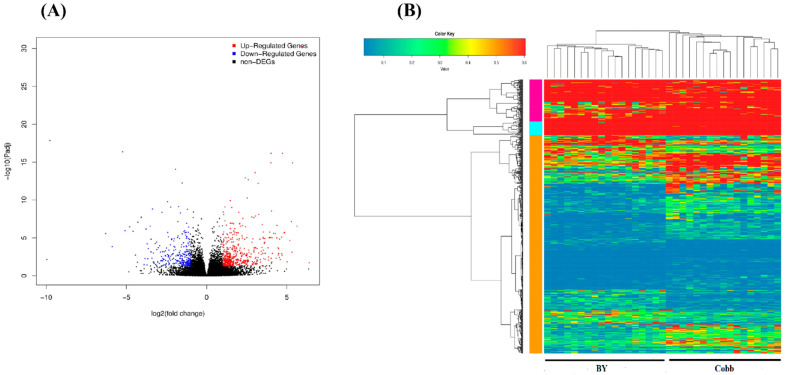
(**A**) Volcano plot showing differentially expressed genes (DEGs) in spleen between BY and Cobb chickens after *Salmonella* infection (log2 FC ≥ 1 and padj < 0.05). (**B**) The heat map based on DEGs of the RNA-seq data.

The DEGs between BY and Cobb chickens were identified by DESeq2. There were 775 significant DEGs in the spleen after ST stimulation (498 up- and 277 down-regulated), as seen in Figure 2A and Supplementary Table S2. Many immune-related genes such as CATH1, AvBD4, BTN3A3, AvBD1, AvBD7, and CNTN3 were significantly upregulated (*p* < 0.01) between BY and Cobb after challenge with *Salmonella* (log2 FC 3.5, 3.53, 4.04, 3.6, 4.37, and 3.04, respectively). The Cluster map of DEGs revealed that the up-regulated more than down-regulated genes in spleen between BY and cobb chickens, as shown in Figure 2B.

### 3.4. Functional Enrichment Analysis of the DEGs

The function analysis of all DEGs was performed using GO and KEGG enrichment. A total of 51 GO terms were enriched and divided into: (i) 24 GO terms under biological process, (ii) 16 GO terms under cellular component, and (iii) 11 GO terms under molecular function, as shown in Supplementary Figure S1. The significantly enriched GO terms related to immune biological processes were mainly involved in immune response (GO:0006955), immune system process (GO:0002376), response to bacterium (GO:0009617), and response to stimulus (GO:0050896), as shown in Table 1.

**Figure 3:**
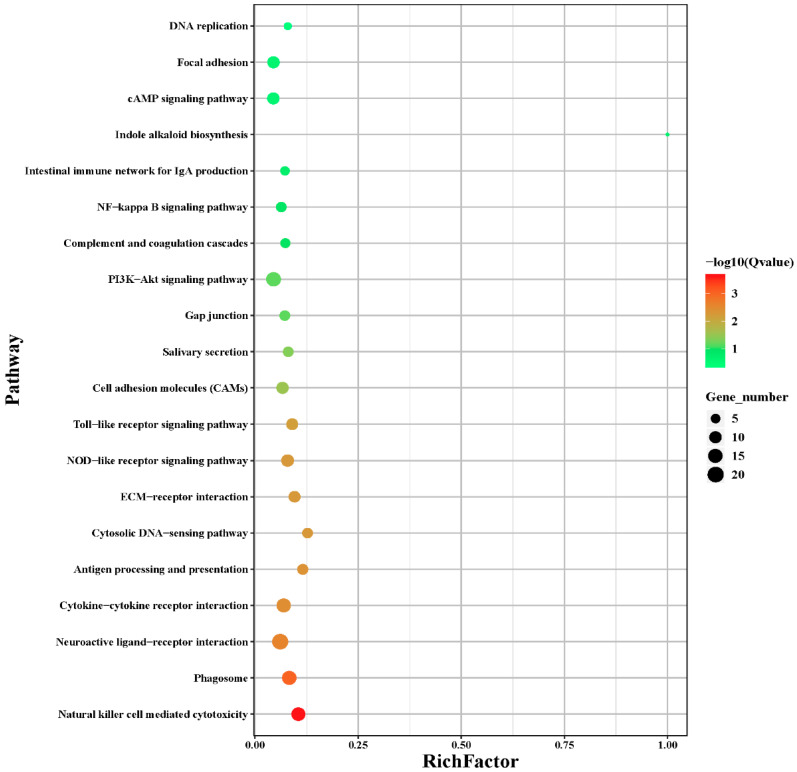
KEGG pathway enrichment analysis. Differentially expressed genes in spleen. y-Axis represents enriched pathways; x-axis represents the rich factor of pathways. Bubble size represents the number of genes, and the color bar represents significance.

After KEGG enrichment analysis, we selected the 20 pathways with the most significant (*p* < 0.05) enrichment. Pathways related to metabolism included Indole alkaloid biosynthesis; pathways related to immune system included toll-like receptor signaling pathways, complement and coagulation cascades, intestinal immune network for IgA production, NOD-like receptor signaling pathways, cytosolic DNA-sensing pathways, and natural-killer-cell-mediated cytotoxicity. In addition, pathways related to cellular processes included focal adhesion, gap junction, and phagosomes; pathways related to signaling molecules and interaction included cell adhesion molecules, ECM-receptor interaction, and neuroactive ligand–receptor interaction, as seen in Figure 3 and Supplementary Table S3.

## 4. Discussion

Changes in the transcriptomes of chickens after *Salmonella* infection have been studied extensively, particularly spleen transcriptomes. Although, few investigations on the differences in the transcriptomes of spleen in local and commercial chickens. The expression patterns of the spleen were compared in BY and Cobb chickens to understand functional and transcriptome alterations after *Salmonella* infection. In addition to clinical signs, the liver bacterial load is considered when evaluating the response of different chicken breeds to ST infection. The spleen expression profiles were determined by transcriptomic analysis. 

In the early phases of *Salmonella* infection in newly hatched chicks, the innate immune system plays a critical role in preventing the spread of the pathogen [27]. The control of bacterial growth is based on host-produced cytokines, but bacterial clearance is dependent on the effective activation of CD4+ T cells, particularly in peripheral immunological organs [28]. 

Significantly altered signaling pathways associated with bacterial–epithelial interactions and immune responses have been identified in the spleen. The significantly altered pathways after ST infection included neuroactive ligand–receptor interaction and cytokine–cytokine receptor interaction, in line with earlier reports [29,30,31]. High doses of *Salmonella* can result in the production of excess amounts of proinflammatory cytokines, or a “cytokine storm”, leading to endotoxin shock or sepsis-related deaths [32,33,34]. Thus, the potential influence of over-expression of inflammatory cytokines due to hypersensitivity response to SE in susceptible birds was also considered in this study.

Several pathways related to the immune system were enriched in Cobb chickens, such as NOD-like receptor signaling pathways, neuroactive ligand–receptor interaction, natural-killer-cell-mediated cytotoxicity, and cytokine–cytokine receptor interaction, in agreement with a previous study [19]. Several immune-related pathways were activated in Cobb chickens, such as neuroactive ligand–receptor interaction, cytokine–cytokine receptor interaction, natural-killer-cell-mediated cytotoxicity, and NOD-like receptor signaling pathways. These findings revealed several signaling pathways controlling the invasion and clearance of *Salmonella*. Moreover, these enriched pathways were shown to be increased by many gene expressions resulting from infection with chicken pathogenic *E. coli* [35,36,37,38].

**Figure 4:**
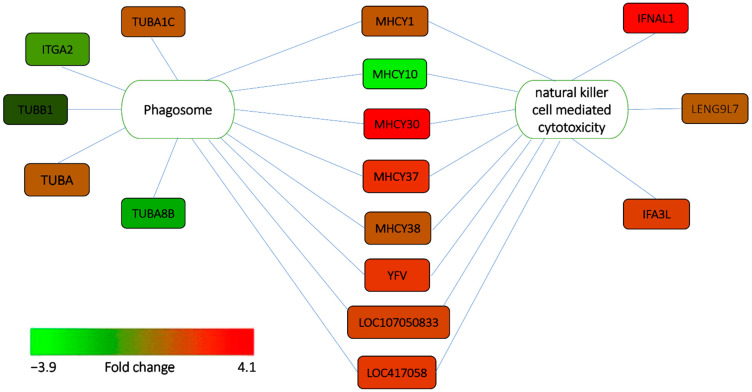
Interaction between signaling pathways and overlapping genes involved in ST infection.

Intriguingly, overlapping genes were found in natural-killer-cell-mediated cytotoxicity, and phagosome and antigen processing and presentation pathways, as shown in Figure 4. This indicates that several signaling pathways may be involved in controlling the invasion and clearance of *Salmonella*. In addition, many genes in these identified pathways are expressed more in response to ST infection. MHC genes in chickens are essential for antigen presentation to T cells, which serve as a vital bridge between adaptive and innate immunity and are associated with resistance to *Salmonella* [39,40].

The hosts have special strategies to combat the infection. This study has enriched natural-killer-cell-mediated cytotoxicity and phagosomes. NK cells are scavenger cells that play a role in innate defense. Their primary function is to recognize and eliminate virally infected, altered, and neoplastic host cells [41,42]. NK cells play an important role in Salmonellosis in the first days of life [43]. 

Upon activation, NK cells release lytic granules containing perforin and granzyme, which are used to lyse target cells [44,45]. NK cells also secrete the cytokine IFNγ to attract other cells to initiate phagocytosis [46]. Heterophils ingest pathogens through a process known as phagocytosis. After ingestion, microorganisms are entrapped in a vacuole called the phagosome, which fuse directly with cytoplasmic granules killing trapped pathogens by releasing antimicrobial peptides and proteolytic enzymes from intracellular granules [47].

**Figure 5:**
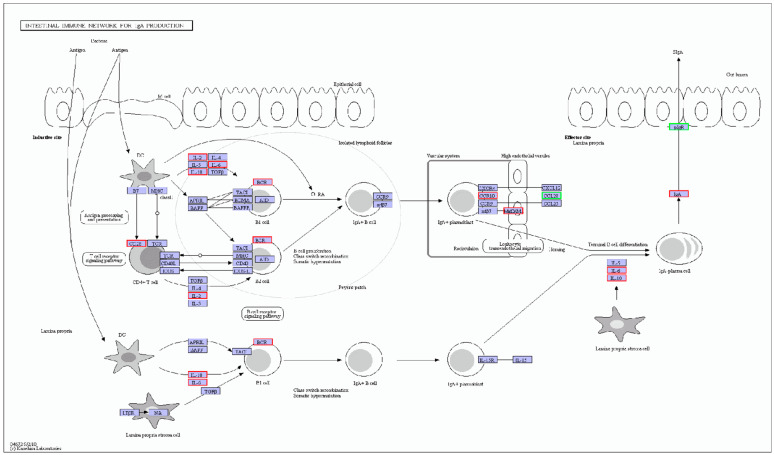
IgA-production-signaling pathway. Green, significantly downregulated DEGs. Red, significantly upregulated DEGs.

The intestinal immune network for IgA production also has been enriched. IgA is a major antibody isotype produced on the mucosal surface and plays an essential role in the immune response of the intestine and prevention of tissue damage during inflammation [19,48,49,50]. Once the intestinal immune system recognizes the invasion of pathogenic microorganisms, IgA is stimulated and transferred into the intestinal lumen [51]. Pathogen-binding IgA has been shown to regulate bacterial movement and protect the host from infection [52]. In addition, IgA inhibits bacterial penetration into epithelial cells by blocking the type III secretion system. [53]. As shown in Figure 5, IgA-production-signaling-pathway-related genes were more expressed in BY chickens.

Earlier studies have shown that macrophages initiate glycolytic upregulation while recognizing microbial ligands. Glycolytic energy promotes antibacterial inflammation and cytokine production [54,55]. After *Salmonella* infection, the spleen is a key immune organ with metabolic variations between BY and Cobb chickens.

## 5. Conclusions

This study aimed to characterize local chickens (BY) and commercial chickens (Cobb) splenic transcriptomes in response to *Salmonella* Typhimurium infection. A total of 775 DEGs were identified between BY and Cobb chicks. DEGs are mostly engaged in the innate immune system’s biological processes, and various pathways, including natural-killer-cell-mediated cytotoxicity, phagosomes, and the intestinal immune network for IgA production, are involved in chicken protection from *Salmonella*. This shows the ability of local chickens to resist *Salmonella* better than commercial chickens, which can pave a road for breeding strategies to improve the resistance of chickens against harmful bacteria in the food industry. 

## Figures and Tables

**Table 1:** Immune-related biological processes identified by gene ontology analysis of DEGs in spleen.

Table	Description	Count	*p*-Value
GO:0006955	immune response	23	0.0001
GO:0009617	response to bacterium	10	0.001
GO:0098542	defense response to other organism	11	0.0013
GO:0002285	lymphocyte activation involved in immune response	6	0.0016
GO:0002376	immune system process	30	0.0058
GO:0006952	defense response	18	0.0067
GO:0051707	response to other organism	13	0.0073
GO:0002520	immune system development	14	0.0129
GO:0050896	response to stimulus	94	0.013
GO:0009607	response to biotic stimulus	13	0.0132
GO:0001775	cell activation	12	0.0205
GO:0032501	multicellular organismal process	73	0.026
GO:0009605	response to external stimulus	24	0.031
GO:0007275	multicellular organism development	56	0.0324
GO:0030154	cell differentiation	43	0.0396
GO:0045087	innate immune response	8	0.0462

DEGs between BY and Cobb chickens were used to identify enriched biological functions (*p* < 0.05).

## Data Availability

The datasets generated during and/or analyzed during the current study are available from the corresponding author on reasonable request.

## Supplementary Materials

The following supporting information can be downloaded at: https://www.mdpi.com/article/10.3390/genes13050811/s1, Figure S1: The enriched GO terms based on the DEGs identified in BY and cobb chicken; Table S1: RNA sequencing Summary; Table S2: Differential Gene Expression data for BY and Cobb chicken; Table S3: The top 20 significant KEGG enriched pathways of BY and Cobb chicken.
