# Supplementary figures and images for: Transcriptomic Analysis of the Spleen of Different Chicken Breeds Revealed the Differential Resistance of Salmonella Typhimurium
Source: Genes (Basel). 2022 May 2;13(5):811. doi: 10.3390/genes13050811 (PMC9142047; doi:10.3390/genes13050811)

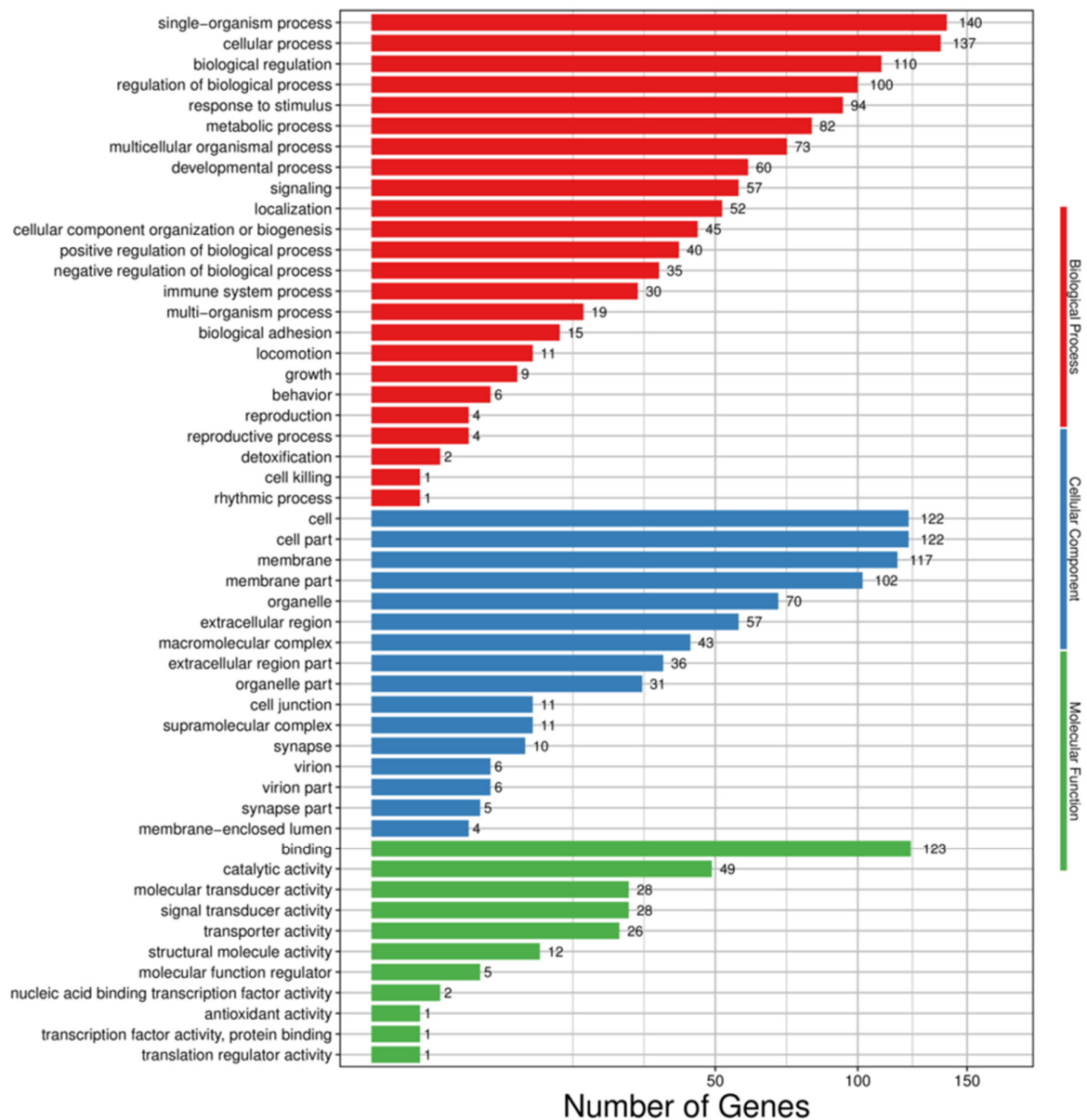

**Figure S1.** The enriched GO terms based on the DEGs identified in BY and Cobb.

Supplement: Supplementary file 1 [file genes-13-00811-s001.zip › Figure S1.pdf]
